# Severity of Congenital Heart Defects Affects Long-Term Somatic Development

**DOI:** 10.1007/s00246-025-03815-7

**Published:** 2025-03-05

**Authors:** Alexandra Kiess, Patricia Bimboese, Ruth Gausche, Christoph Beger, Christof Meigen, Mandy Vogel, Roland Pfäffle, Ingo Dähnert, Wieland Kiess

**Affiliations:** 1 Department of Child and Adolescent Medicine, Section of Pediatric Cardiology, University Hospital Jena, Am Klinikum 1, 07747 Jena, Germany; 2 Department of Pediatric Cardiology, Faculty of Medicine, Heart Center Leipzig, Leipzig University, 04289 Leipzig, Germany; 3 Growth Network CrescNet, Leipzig University, 04103 Leipzig, Germany; 4 LIFE-Child-Leipzig Research Center for Civilization Diseases, Leipzig University, 04103 Leipzig, Germany; 5 Hospital for Children and Adolescents, Leipzig University, 04103 Leipzig, Germany; 6 German Center for Child and Adolescent Health (DZKJ), Partner Site Leipzig/Dresden, Leipzig, Germany

**Keywords:** Congenital heart disease, Growth, Somatic development, Long-term growth, Severity of congenital heart disease

## Abstract

Treatment options and therefore general health in children with congenital heart defects (CHD) improved tremendously over the last decades. Growth is an important marker to evaluate healthy development, especially in patients with chronic diseases. This study aimed to evaluate growth in children with CHD compared to their healthy peers. A retrospective, longitudinal analysis of standardized measurements of height, weight, and head circumference of children with CHD aged 0 to 18 years (*n* = 18,591) was conducted based on data provided by the German pediatrician network CrescNet. These data were compared to the data of healthy children from the same region. Inclusion was based on the ICD-10 codes for CHD; patients with other growth-altering diseases or medications were excluded. CHD were grouped into mild, moderate, or severe according to their severity. The severity of the CHD influences the height, final height, weight, and head circumference of affected children at birth and in long-term growth. Children with more severe CHD are smaller (− 1 to − 0.7 SDS), lighter (− 1 SDS), and have smaller head circumferences (− 1 SDS) than their healthy peers and children with milder CHD. In a subgroup analysis of children with Down’s syndrome (T21), children with T21 and CHD showed significantly smaller measurements than their peers without CHD. In this subgroup, only the presence but not the severity of the CHD mattered. Despite overall good outcomes and better survival rates, growth as a marker of healthy development in children with CHD may be still impacted and should be closely monitored to offer early intervention.

**Supplementary Information:**

The online version contains supplementary material available at 10.1007/s00246-025-03815-7.

## Introduction

Diagnostics and treatment options for children with congenital heart defect (CHD) have improved tremendously over the last decades. Therefore, the focus in CHD care is moving progressively from improving survival rates to improving long-term outcomes. Somatic development as an important marker of healthy development in childhood and adolescence is one of the benchmark measurements, especially in patients with chronic diseases [1]. Somatic development can also reflect on neurodevelopment. Microcephaly and low birth weight are some of the factors attributed to increased risk of neurodevelopmental impairment [2]. A recent review on growth and risk of adverse neurodevelopmental outcomes in children with CHD showed a correlation between head circumference at birth and performance in neurocognitive assessments in later childhood. Head circumference later in life was associated with motor delays, impaired fine-motor skills, and lower psychomotor developmental indices [3].

Poryo et al. described the somatic development in children with CHD during the first 2 years of life with data derived from the German Competence Network for CHD in 2018. Their study found that children with severe CHD had lower heights, weights, and head circumferences compared to the German reference values. Furthermore, no catch-up growth was noted until the age of two years [4].

The aim of our study was to complement the existing data by evaluating long-term growth in children with CHD to describe predictors of altered somatic development with a special focus on CHD severity. We used a general population-based approach to collect data using the CrescNet database [5]. CrescNet contains data on almost one million German children, of whom roughly one percent had at least one ICD-10 code indicating the presence of CHD.

Since not only CHD but also syndromic diseases influence somatic development, we excluded children with known syndromes from the general analysis. To further investigate growth in children with CHD and syndromic diseases, we chose Down’s syndrome (T21) as the most common syndrome in our cohort. We compared the growth data of children with T21 and CHD with their peers without CHD and set this data in relation to the specific percentiles for children with Down’s syndrome [6].

## Material and Methods

### Population Description

Health data, including anthropometric data and ICD-10 codes, were obtained from the CrescNet database, a national Internet-based database‍ [5]. The network was established at the University Children’s Hospital Leipzig in 1998 for the early detection of growth disturbances in the pediatric population [7]. Anthropometric data collected at medical checkups and visits from birth through adolescence are entered by several hundred pediatricians across Germany. The database currently contains anthropometric data from almost one million children and adolescents. The epidemiological work of the CrescNet was approved by the ethics committee of the Leipzig University and is registered at the Clinical Trials Registry (Clinicaltrials.gov; identifier NCT03072537).

All children who contributed data between 1999 and 2023, whose caregivers gave written informed consent to data usage for research, and who had at least one coding code for CHD from the ICD-10 catalog Q20–Q26 were included (*n* = 17,834). Additionally, children with T21 and without CHD were included (*n* = 757) as a comparison group for children with CHD and T21. Their data were only used for the specific analysis with their peers with T21 and CHD. Most children attended several visits with their pediatrician, including collection of their anthropometric data (*n* = 86,544 and *n* = 3020 for T21). We retrospectively collected documented measurements of body height/length, weight, and head circumference at different ages, as well as gestational age and parental heights, when available, for each individual child. We excluded subjects exposed to potentially growth-altering medications (Suppl. Table 1) and with diagnoses, especially genetic disorders, being known to impact growth (Suppl. Table 2).

We classified the CHD into severity groups according to the ICD-10 codes (Suppl. Table 3). For most defects, we utilized the classification used by Poryo et al. [4]. For others, we chose the most reasonable group through clinical judgment when the disease severity was not clearly encoded through ICD-10 codes. We then selected exactly one ICD-10 code for each subject, prioritizing the highest severity according to our beforementioned classification (Suppl. Table 4). The resulting severity groups of subjects are described in Suppl. Table 5 with respective measurements of body height, weight, BMI, and head circumference, as well as birth data.

### Statistics

We calculated standard deviation score (SDS) values based on the reference data from the KiGGS study [8] and Voigt et al. [9] for gestational age-corrected birth measurements. Zero SDS refers to the population mean. Target height was calculated according to Hermanussen et al. [10] Near-final age was set to age in years ≥ 18 for males and ≥ 16 for females. Outcomes were compared at predefined ages (2, 6, 10, 14, and 16 years of age). The respective age groups were defined as 2 ± 0.5, 4 ± 0.5, etc. Significance level was set to 5%. Calculations were performed with R version 4.2.2 [11].

Age trends were modeled stratified by severity and sex applying generalized additive models (GAM) [12, 13], including a cubic spline in age for smoothing [14]. We introduced weights to account for repeating observations. For all relevant outcomes, the distributions of SDS were compared to the respective distributions in the healthy reference population and between each other using Student’s *t* test [15]. Proportions of children having low height, weight, BMI, or head circumference (microcephaly) at birth were compared between groups using risk ratios (RR) and Fisher’s exact test [16].

## Results

### Birth Measurements

**Fig. 1:**
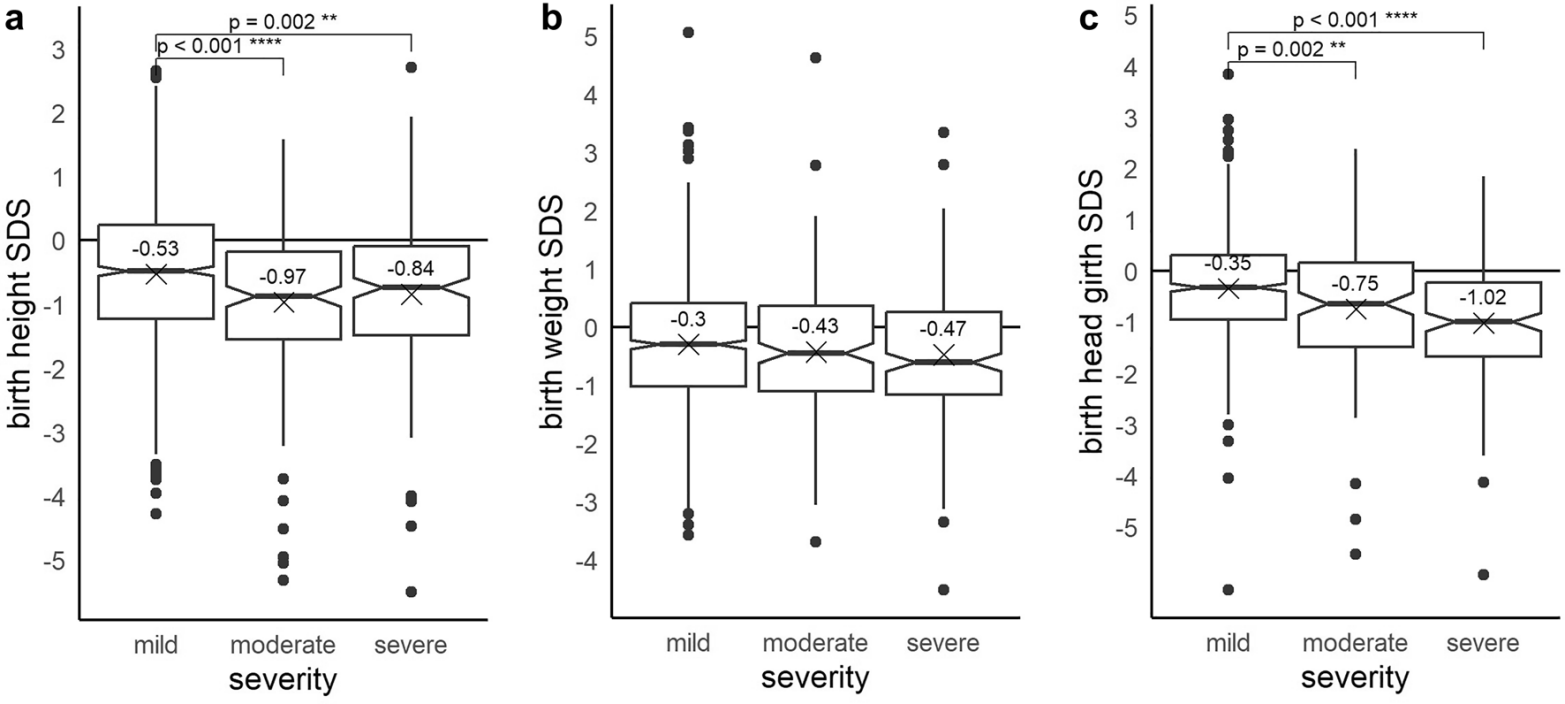
**a**–**c** Boxplots of gestational age-corrected birth height (**a**), weight (**b**), and head circumference (**c**) SDS according to Voigt et al., grouped by CHD severity. Brackets with stars indicate significant differences between groups. Head circumference and height measurements in neonates with moderate and severe CHD were significantly lower than in neonates with mild CHD and the reference population

Gestational age-corrected birth measurements were available for 1226 (height), 1300 (weight), and 936 (head circumference) children. Neonates with CHD had a significantly lower gestational age-corrected birth height (− 0.64 SDS, *p* < 0.001), weight (− 0.35 SDS, *p* < 0.001), and head circumference (− 0.53 SDS, *p* < 0.001) than the reference population. The effect was greater in children with moderate and severe CHD than in children with mild CHD. The biggest influence was seen in head circumference development. We found that children with severe CHD had an SDS of − 1.02 (*p* < 0.001) and children with moderate CHD an SDS of − 0.75 (*p* = 0.002) compared to the head circumference in the reference population (Fig. 1). Microcephaly at birth was seen in 7.8% of the children with mild CHD, compared to 17.4% (RR = 2.24, Fisher’s test: *p* = 0.001) with moderate and 21.7% (RR = 2.80, *p* < 0.001) with severe CHD. The impact on birth height and weight was similar. 13.4% of the children with CHD were born small for gestational age (height or weight below − 2 SDS). This effect was greater in children with moderate (17.5%, RR = 1.51, *p* = 0.035) and severe CHD (16.4%, RR = 1.41, *p* = 0.072) than in children with mild CHD (11.6%).

### Long-Term Height

**Fig. 2:**
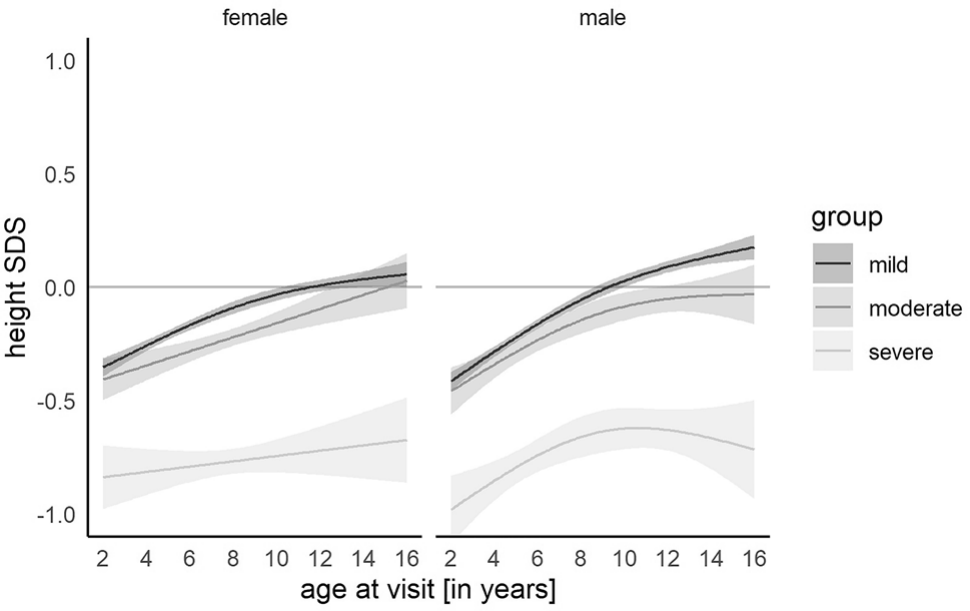
Fitted generalized additive model of height SDS for children with mild, moderate, and severe congenital heart defect showing a significantly lower height in children with severe heart defects. The gray shades represent the 95% confidence interval

Our data also demonstrated an impairment of long-term height in children with severe CHD. Children with mild and moderate CHD initially had lower heights than the reference population but experienced catch-up growth and eventually reached the heights of the reference population. Children with severe CHD remained smaller than their peers. There was no difference between males and females (Fig. 2).

We analyzed age and sex subgroups to investigate which groups were at the highest risk of growth impairment. At the age of two years, children with severe CHD were smaller than their healthy peers, with a deviation of − 0.84 SDS (ci [− 0.98; − 0.7]) in female and − 0.98 SDS (ci [− 1.13; − 0.83]) in male children. The differences in height SDS were less prominent in children with mild (females − 0.35 SDS (ci [− 0.39; − 0.31]), males − 0.41 SDS (ci [− 0.5; − 0.32])) and moderate (females − 0.41 SDS (ci [− 0.5; − 0.32]), males − 0.46 SDS (ci [− 0.56; − 0.35])) CHD. The mean height of adolescents with mild and moderate CHD improved and approached the height of healthy children at later ages (up to 16 years). Only adolescents with severe CHD remained significantly smaller, with − 0.67 SDS (ci [− 0.86; − 0.49]) for females and − 0.72 SDS (ci [− 0.93; − 0.5]) for males.

### Near-Final Height And Parental-Corrected Near-Final Height

Measurements of near-final height (defined as height at age > 18 years for males and > 16 years for females) were available for 1150 children. Adolescents with moderate and severe CHD had a significantly lower near-final height (− 0.22 SDS, *p* = 0.039; and − 0.62 SDS, *p* < 0.001, respectively) than those with mild defects (0.05 SDS) and the normal population.

**Fig. 3:**
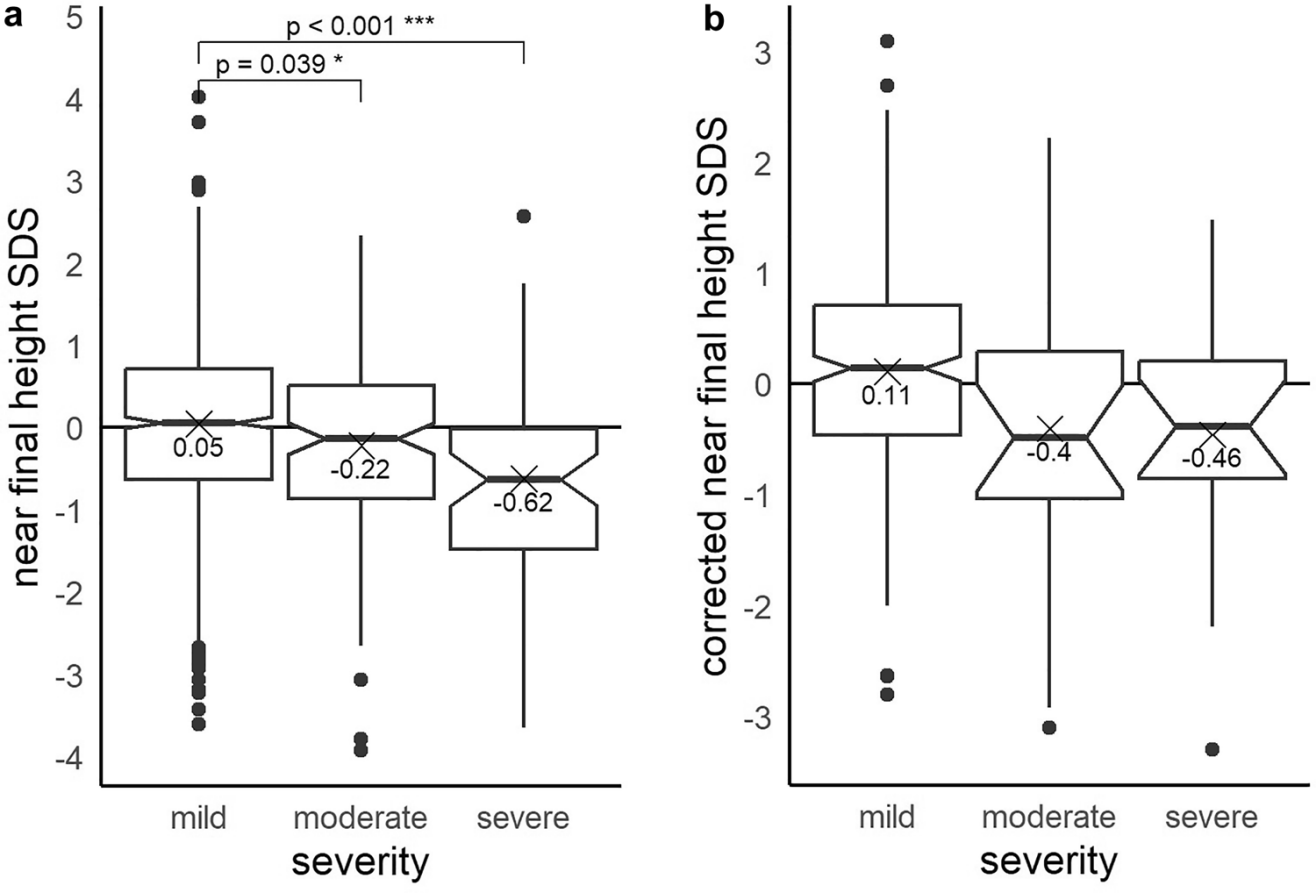
**a**, **b** Boxplots of near-final height SDS with and without parental height correction for children (set to age > 18 years of age for males and > 16 years of age for females) with mild, moderate, and severe CHD showing a significantly lower near-final height in children with moderate and severe heart defects (**a**). Parental-corrected near-final height showed a similar trend but did not reach significance due to the small number of cases (**b**)

For 304 children, the heights of both parents were available in addition to the final heights. After adjusting for both parents’ heights, the observed effects in this considerably smaller group were similar but did not reach statistical significance, most likely due to the small group size (Fig. 3).

### Weight and BMI

**Fig. 4:**
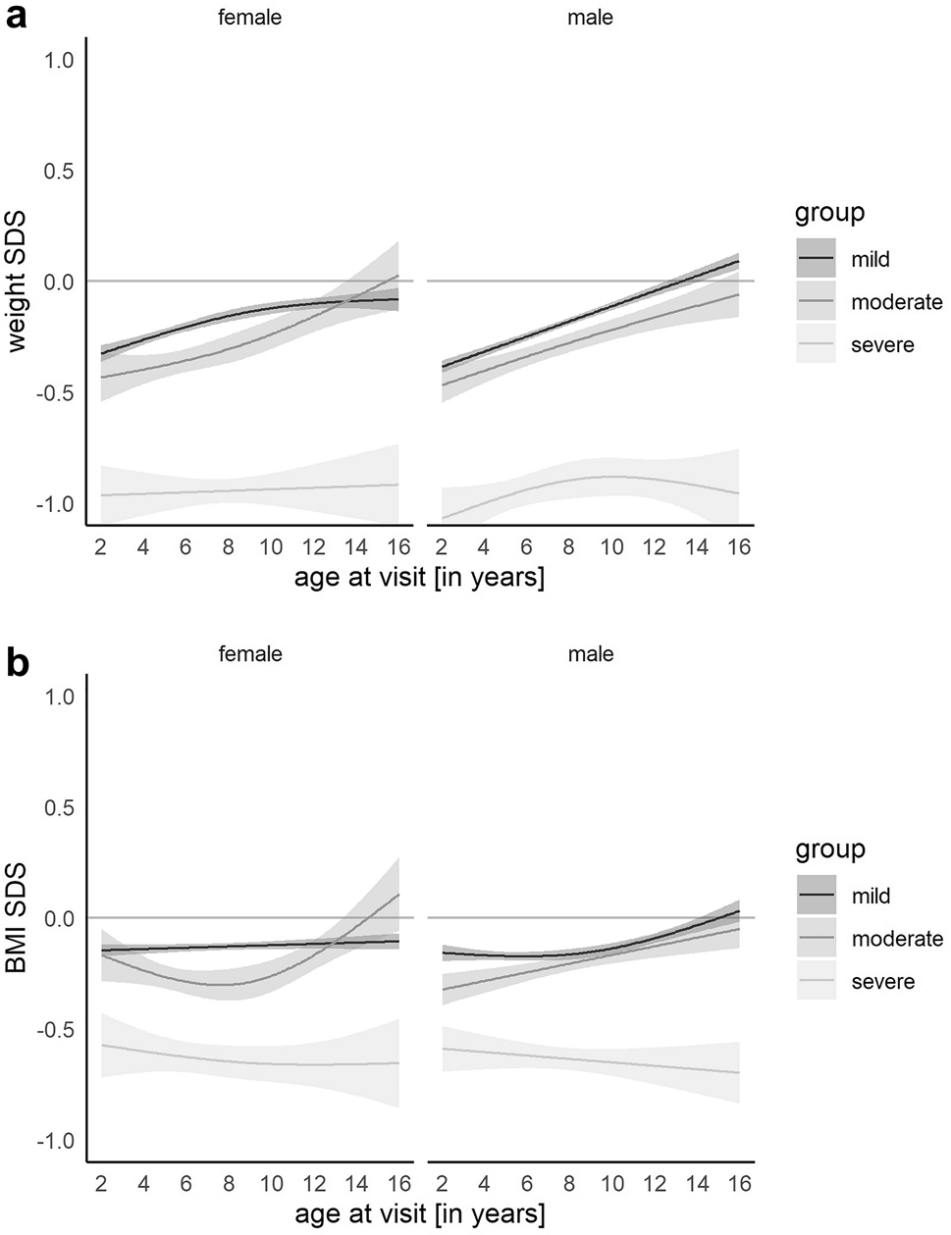
**a**, **b** Model of weight (**a**) and BMI (**b**) SDS for children with mild, moderate, and severe congenital heart defect showing a significantly lower-weight SDS and BMI SDS in children with severe CHD compared to the reference population and children with mild and moderate CHD. The gray shades represent the 95% confidence interval

The impairment of weight and BMI in children with CHD also correlated with the CHD severity. During the first years of life, the weight and BMI SDS were significantly lower in all CHD groups than in the reference population. From two years of age up to an age of 16, children with severe CHD had an approximately 1 SDS lower weight SDS than the reference population. In contrast, we observed an increase in weight SDS for both sexes in the mild and moderate subgroups, starting at the age of two years with approximately − 0.4 SDS reaching 0 SDS during adolescence. Similarly, the BMI SDS in children with severe CHD was lower compared to children with mild CHD and to the reference population (− 0.5 to − 0.7 SDS, *p* < 0.001) regardless of sex and age (Fig. 4).

### Head Circumference

**Fig. 5:**
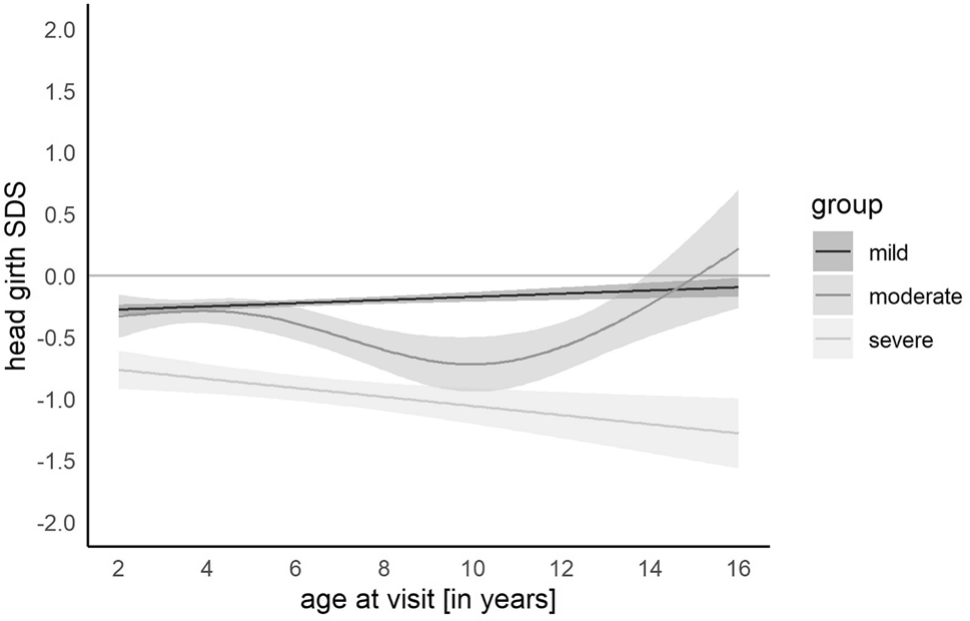
Model of head circumference SDS for children with mild, moderate, or severe congenital heart defect showing a significantly lower head circumference in children with severe heart defects. The gray shades represent the 95% confidence interval

We found corresponding results for head circumferences for children with CHD. Children with severe CHD exhibited smaller head circumferences compared to their healthy peers and children with moderate and mild CHD (approx. − 1 SDS). No sex differences were found. Increasing age was linked to a catch-up head growth in most children with mild and moderate CHD, whereas the head circumference of children with severe CHD stayed significantly below the reference values (Fig. 5).

### Differences in Growth Between Specific Moderate and Severe Heart Defects

To further investigate whether different moderate and severe congenital heart defects have a distinct influence on growth, we analyzed specific defects separately. This included severe defects such as univentricular hearts (UVH), transposition of the great arteries (TGA), and tetralogy of Fallot (TOF), as well as moderate CHD, such as coarctation of the aorta (CoA) and atrioventricular septal defect (AVSD). Results are listed in Supplementary Table 6.

In general, children with UVH and TOF were significantly shorter than children with moderate CHD, TGA, and the reference population. At the age of 2 years, children with UVH and TOF had a significantly lower height SDS (UVH: − 1.17 SDS, ci [− 1.39; − 0.94]; TOF − 1.06 SDS, ci [− 1.35; − 0.78]). Both groups showed a slow but steady increase in their height SDS during childhood, resulting in an SDS of − 0.8 (UVH, ci [1.05; − 0.54]) and − 0.69 (TOF, ci [− 1.04; − 0.35]) at the age of 12 years. Children with TGA, COA, and AVSD showed a milder alteration of their height of around − 0.2 to − 0.6 SDS compared to the reference population.

The analysis of weight SDS showed similar, even more pronounced results. Children with UVH and TOF were significantly lighter (between − 0.99 and − 1.37 SDS) than children with moderate diseases and TGA (around − 0.5 SDS). Children with TOF showed a weight gain from − 1.32 to − 0.99 SDS, whereas children with UVH had no significant catch-up of their weight SDS (− 1.13 SDS at the age of 2 years to − 1.18 at the age of 12 years).

Children in the CHD groups showed lower BMI than the reference population. Children with TOF and UVH were again the most affected groups. In children with TOF, the BMI SDS was around − 0.9 with only a slight decrease with advancing age, while in their counterparts with UVH, the BMI SDS decreased with age from − 0.5 (2 years) to − 0.85 SDS (12 years).

The results for head circumference were compromised by the limited sample size, especially in older children. Children with TOF showed the highest impairment in early life with a catch-up growth, whereas in children with UVH, the measurements in early childhood were less impacted but no catch-up growth was noted. Interestingly, children with TGA also showed smaller head circumferences across childhood, increasing from − 0.65 SDS (ci [− 0.97; − 0.33]) at 2 years to − 0.53 SDS (ci [− 1.15; − 0.1]) at the age of 12 years despite having height and weight SDS closer to the reference population (− 0.19 to − 0.5 SDS).

### Growth in Children with Down’s Syndrome With and Without CHD

CHD can occur in conjunction with syndromic diseases. Children with syndromic diseases often display failure to thrive or a short stature. Special growth percentiles for affected children were created for some syndromes, for example, for children with Down’s syndrome (T21) by Zemel et al. [6]. In the cohort used by Zemel et al., approximately half of the children had a CHD diagnosis. To further investigate whether the influence of the syndromic disease or the CHD is more relevant for growth, we analyzed the data of 757 children with Down’s syndrome from our database. Of those children, 522 did not have any CHD diagnosis (T21/no-CHD). Due to the low number of moderate and severe CHD cases and no significant differences between the severities in the baseline analysis, we combined the CHD severity groups ‘mild,’ ‘moderate,’ and ‘severe’ (as used above for children without T21 for our patients with T21 (T21/CHD)). Descriptive data are shown in Supplementary Table 7.

In our cohort, the children with T21 were generally slightly taller than the children from the Zemel cohort with no significant differences between the sexes. Comparing the heights of 2-year-old children stratified by CHD/no-CHD, we found no difference between the reference population and the T21/CHD group (+ 0.06 SDS, *p* = 0.33) but significantly higher height SDS in the T21/no-CHD children (+ 0.57 SDS, *p* < 0.001). The comparison at 16 years of age showed no significant differences between both groups (− 0.01 SDS in T21/CHD, *p* = 1.00) and compared to the Zemel cohort (both + 0.13 SDS, *p* = 0.10).

Children with T21 in our cohort were also lighter than in the reference cohort. Children with T21/CHD showed lower weight SDS than the Zemel cohort and their peers with T21/no-CHD. Levels of significance were only reached at 2 years of age (T21/CHD: − 0.34 SDS, *p* < 0.001, T21/no-CHD: − 0.10 SDS, *p* = 0.002) and 6 years of age (T21/CHD: − 0.28 SDS, *p* < 0.001, T21/no-CHD: − 0.08 SDS, *p* < 0.001) compared to the Zemel cohort.

The BMI SDS of our T21 cohort was significantly lower than in the reference population by Zemel et al. The difference was more pronounced in children with T21/CHD and in younger age. Children at the age of 2 years had a BMI of approximately − 0.7 SDS, again without significant differences between the CHD and no-CHD group (+ 0.01 SDS, *p* = 0.80). We found a steady increase of BMI SDS in both groups up to 16 years of age (T21/CHD: − 0.17 SDS, *p* = 0.03, T21/no-CHD: 0.00 SDS, *p* = 0.99), with no significant difference between both groups (− 0.17 SDS, *p* = 0.59).

**Fig. 6:**
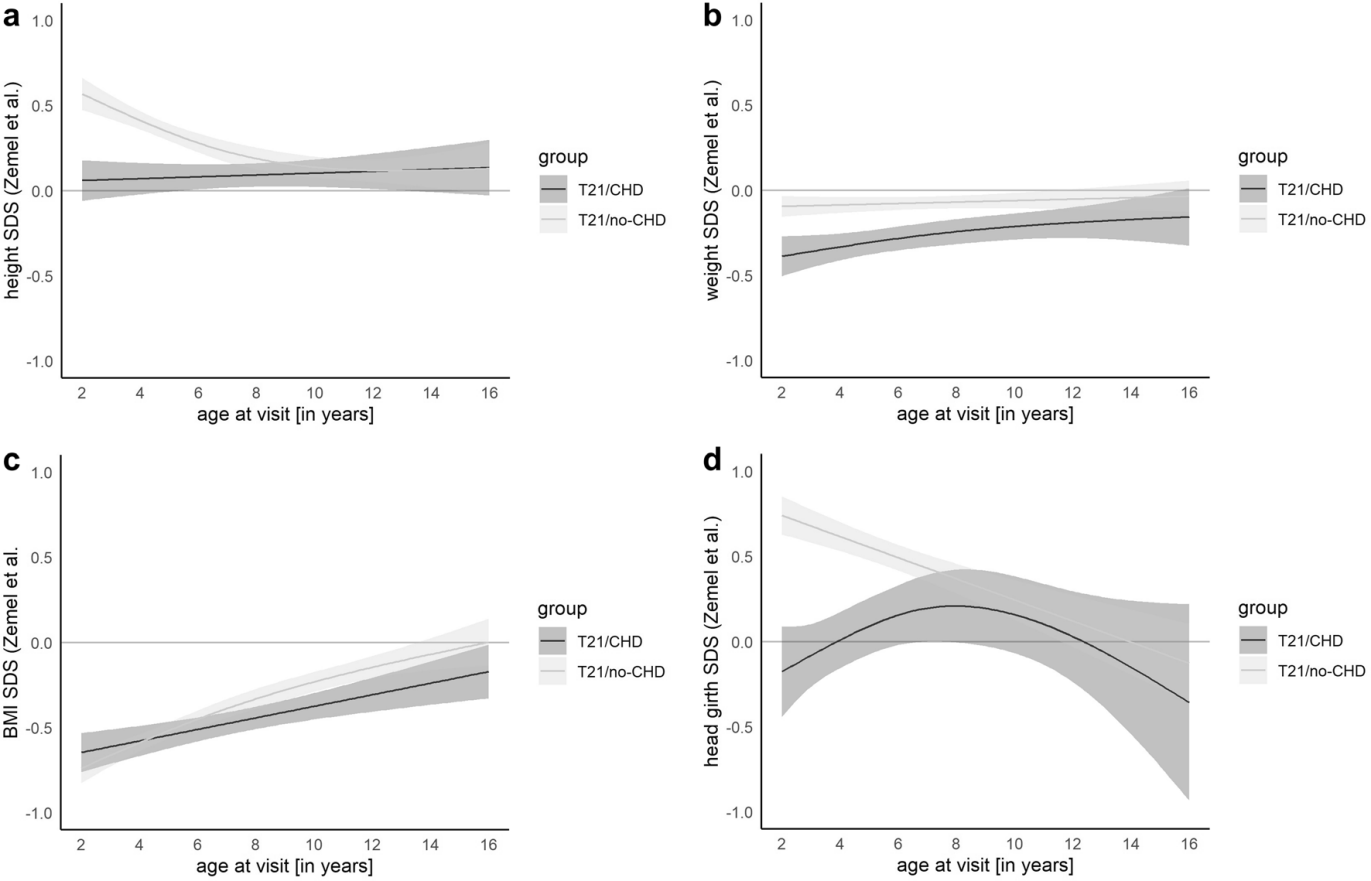
Model of height (**a**), weight (**b**), BMI (**c**) and head circumference (**d**) SDS for children with Down’s syndrome and with and without congenital heart defect. Our data show an influence of the presence of CHD on growth in children with Down’s syndrome reaching levels of significance in head circumference, BMI, and weight where children with CHD showed lower median SDS

The presence of CHD had the greatest influence on head circumference in our Down’s population. In our cohort, children with T21/CHD had significantly smaller head circumference SDS than their peers T21/no-CHD at the age of 2 years (− 0.18 vs. 0.74, *p* < 0.001). This observation became less pronounced and eventually insignificant with advancing age. Interestingly, the head circumference of the Zemel cohort was not different from the children in our cohort with T21/CHD at the age of 2 years (*p* = 0.19), but again smaller than T21/no-CHD children (*p* < 0.001). *(*Fig. 6*).*

## Discussion

### Birth Measurements, Including Head Circumference, are Affected by the Severity of CHD

To date, several studies have shown that neonates with CHD are at risk of having smaller head circumferences than the healthy population. Poryo et al. reported birth measurements of 1093 children with CHD. Microcephaly, defined as a head circumference < 3rd percentile, was found in 20.9% of them. The percentage in children with severe defects (26.2%) was higher than in children with moderate (23.4%) or mild defects (18%) [4]. Our study could confirm these findings with data on 936 neonates, showing slightly lower microcephaly rates (CHD severe: 21.7%; moderate: 17.4%, mild: 7.81%).

The birth weight in our cohort was only mildly affected by the presence of CHD (severe − 0.47, moderate − 0.43, and mild − 0.30 SDS). A more pronounced impact was seen for height. Children with more severe CHD were more affected (severe − 0.84, moderate − 0.97, mild − 0.53 SDS). 13.4% of the children with CHD were born small for gestational age (height or weight below − 2 SDS), with a greater effect in children with moderate (17.5%) and severe CHD (16.4%) than in children with mild CHD (11.6%). Aliasi et al. reviewed 23 publications and found a SGA prevalence of 16.0% (defined as birth weight < 10th percentile) in CHD patients. Their subgroup analysis of major CHD showed a similar risk of 16.2% [17]. The difference with our findings could be due to different definitions of SGA (3rd vs. 10th percentile vs. − 2 SDS), but also the heterogeneous design and patient cohorts of the reviewed studies.

### Catch-Up Growth Remains Incomplete

Poryo et al. analyzed growth of children with CHD in their first two years of life and noted no catch-up growth in children who underwent cardiac surgery. The median body weight and length/height ranged between the 10th and 50th percentiles. Daymont et al. confirmed the incomplete catch-up growth. It remained incomplete in their cohort of children requiring surgical repair until 36 months of age [18]. Those findings are congruent with our data. Catch-up growth started later in childhood but remained partially incomplete, especially in children with more severe CHD. Growth patterns of children with different severe CHD seem to be strongly impaired. A pronounced growth restriction even in later years is stated for children with TOF and UVH which is congruent to our data. Li et al. reported a higher prevalence of body height and weight restriction in children with TOF aged 1–5 years rather than in infancy [19].

Children with UVH who undergo univentricular palliation are among the better-studied subgroups. Van den Eynde et al. reviewed nineteen studies with a total of 2006 children. They found reduced *Z-*scores for height and weight from birth to the interstage period and a partial catch-up growth of about 50% following the Glenn procedure. The *Z-*scores for height were still lower in adolescents after complete univentricular palliation. The by-then normalized weight resulted in an increased BMI in those children [20]. Children in our cohort showed a similar reduced height as youths, but weight and BMI remained also lower with median SDS below − 1.0 and − 0.5 SDS. This could be simply due to our smaller cohort, but also other influences like different timing of surgery, different approaches in heart failure prevention, and different lifestyle recommendations need to be considered.

### CHD Severity Affects Long-Term Growth

We could demonstrate that the severity of CHD has a negative impact on long-term growth. Children with severe CHD are more likely to be smaller, lighter, and have a smaller head circumference than their peers with no, mild, or moderate disease. Our data are consistent with the findings of other groups, which mostly focused on early development. For example, our long-term follow-up data showed a median head circumference of around − 0.77 to − 1.28 SDS in severely affected children compared to children with mild to moderate CHD with a median head circumference of approximately − 0.4 SDS. This confirms similar parameters reported by Poryo et al., with median head circumferences between the 10th and 25th percentiles for severely affected children in comparison to medians between the 25th and 50th percentiles in moderately affected children [4]. Another single-center study found that the risk of having a height < − 2 SDS was around 3% in patients with mild CHD, three times higher for moderate CHD and four times higher for severe CHD, compared to about 1% without CHD [21]. Interestingly, there are also data with contrary results. In a Swiss study, CHD patients at 10 years had normal *Z*-scores for height and BMI, but the *Z*-scores for weight and head circumference were significantly lower (− 0.38 and − 0.71) [22]. This could be due to the different study end point at 10 years and the study composition without stratification into severity.

Not only the CHD severity but also the kind of the lesion seem to matter. Further subgroup analysis of our cohort showed that children with UVH and TOF had the most abnormal growth patterns, a finding that was also seen by Aguilar et al. [23].

### Other Risk Factors for Insufficient Growth in Children with CHD

Along with the severity, other risk factors have been reported to influence growth. Age was reported by several studies as one of the risk factors. However, this factor remains inconclusive or variable between different study cohorts. Talassi et al. reported an age of < 1 year among the growth impairing factors. The group further identified fewer appointments, absence of surgery, and low family income [24]. Additional reported risk factors for lower height at age 10 [22] and 12 [25] years include multiple intraoperative bypass runs, a co-existing feeding disorder diagnosis [25] and length of hospital stay at first bypass run [22]. The latter two were also associated with lower weight. Interestingly, the length of hospital stay was not associated with head circumference [22]. Van den Eynde et al. published factors with a positive influence on growth in their review on UVH patients, including timing of surgery, aggressive nutritional and complication management, as well as obesity prevention programs for adolescents [20].

Dalili et al. reported large left-to-right intracardiac shunts, pulmonary hypertension, and cyanosis as well as being female as risk factors in Iranian children with hemodynamically relevant CHD [26]. Another study compared children with cyanotic and acyanotic CHD and affirmed cyanosis as a risk factor [27]. A study by He et al. added preoperative anemia, left ventricle dysfunction, younger age, more complex CHD, lower birth weight, and an underlying genetic syndrome to the risk factors for low weight or height [28].

### Growth in Children with Down’s Syndrome and CHD

In our cohort, children with T21 and CHD were slightly lighter and had lower BMI, but no constant significant difference in height, compared to the Zemel reference population. Interestingly, the children with T21 and no-CHD from our cohort were taller than the reference population and showed higher head circumference until the age of 10 years. The head circumferences of our patients with T21/CHD were smaller than the measurements from their no-CHD peers from our cohort, but not smaller compared to the reference population. These differences could be explained by the cohort composition of the reference population consisting of children with and without CHD (53 vs 47%) with a prematurity rate of about a quarter of those children (gestational age < 37 weeks). Further potential explanations for the varying results are differences in socioeconomic background, medical care, dietary habits, and frequency of overweight in the general population.

### Advantages and Drawbacks

Studies in children with CHD are generally complicated due to the heterogeneity of the diseases and the different treatment approaches varying by centers, including different heart failure treatment strategies and nutrition management as well as significantly progressing surgical concepts over the last decades. The complexity of growth and somatic development on the other hand complicates the multilayer puzzle.

Our population-based approach allowed us to include a high number of children. Therefore, we were able to perform several subgroup analyses and generate long-term follow-up data. The cost of the population-based approach is the potential inaccuracy of the diagnosis based solely on ICD-10 coding to include affected children and partially incomplete datasets that cannot exclude influences of other growth-altering factors, for example, shorter gestation in some children. However, through our approach, we were able to compare our dataset to a heterogeneous reference population from the same region and approximately same data collection period.

## Conclusion

Over the last years, we moved from solely ensuring the survival of the child with CHD to optimizing long-term outcomes as a quality indicator for CHD management. Growth and somatic development will be important markers of successful treatment of patients with CHD. We could demonstrate that both, birth measurements and long-term somatic development, are still affected in children with CHD depending on the CHD severity. Somatic development of children with Down’s syndrome is also affected by co-existing CHD especially in the first years of life. Our study supports existing data on impaired growth in CHD patients and adds interesting data from a population-based approach on long-term development in children with CHD, including tendencies for near-final height and parental-corrected near-final height, and for different moderate and severe heart defects like children with univentricular hearts.

Our study highlights that growth in CHD patients should be closely monitored to intervene early and offer potential treatment options.

## Supplementary Information

Below is the link to the electronic supplementary material.

Supplementary file1 (DOCX 46 KB)

## Data Availability

De-identified data collected for this study, a data dictionary and statistical analysis plan are available from the corresponding author on reasonable request. Aggregated data are included in the supplementary files of this article.

## Abbreviations

AVSD: Atrioventricular septal defect
BMI: Body mass index
CHD: Congenital heart defect
CoA: Coarctation of the aorta
GAM: Generalized additive model
ICD-10: International statistical classification of diseases and related health problems (10th revision)
RR: Risk ratio
SDS: Standard deviation score
T21: Trisomy 21
TGA: Transposition of the great arteries
TOF: Tetralogy of fallot
UVH: Univentricular heart
