# Supplementary material for: Severity of Congenital Heart Defects Affects Long-Term Somatic Development
Source: Pediatr Cardiol. 2025 Mar 5;47(2):642–51. doi: 10.1007/s00246-025-03815-7 (PMC12855346; doi:10.1007/s00246-025-03815-7)
Supplement: Supplementary file 1 — Supplementary file1 (DOCX 46 KB) [file 246_2025_3815_MOESM1_ESM.docx]

# Supplements

# Tables

Suppl. Table 1: Medication (drug name and ATC code) usage that led to exclusion of a subject from analysis.

| Medication | ATC code |
| --- | --- |
| Somatotropin Hormone (STH) | H01AC01 |
| Oral Glucose tolerance test (oGTT) | n.a. |
| Testosterone | G03BA03 |
| Hydrocortisone | H02AB09 |
| Enantone (Leuprorelinacetate) | n.a. |
| Fludrocortisone | H02AA02 |
| Carbimazole | H03BB01 |
| Methimazole | H03BB02 |
| Increlex (Mecasermin) | H01AC03 |
| Octreotide | H01CB02 |
| Diazoxide | V03AH01 |
| Ethinylestradiol (EE2) | L02AA03 |
| Presomen (conjugated oestrogens) | n.a. |
| Desmopressin | H01BA02 |
| Gestagene | n.a. |
| Gonadotropin-releasing hormone (GnRH) | n.a. |
| Gonadotropin-releasing hormone (GnRH) agonist | n.a. |
| Glucocorticoide | H02AB |
| Oxandrolone | A14AA08 |
| Methotrexate | L04AX03 |
| Vosoritide | M05BX07 |
| Sapropterin (Tetrahydrobiopterin) | A16AX07 |
| Neridronate | n.a. |
| Somatrogon | H01AC08 |
| Burosumab | M05BX05 |

Suppl. Table 2: Underlying conditions (diagnosis and ICD-10-GM codes) that led to exclusion of a subject from analysis. Regular expressions are provided that can match multiple ICD-10-GM codes.

| Exclusion diagnosis | ICD-10-GM codes |
| --- | --- |
| Elli van Creveld Syndrome | Q77.* |
| Other specified congenital malformation syndromes affecting multiple systems | Q78.* |
| DiGeorge Syndrome (22q11 deletion syndrome) | Q82.1 |
| Phakomatoses, not elsewhere classified | Q85.* |
| Congenital malformation syndromes due to known exogenous causes, not elsewhere classified | Q86.* |
| Noonan Syndrome | Q87.1 |
| Cornelia de Lange Syndrome | Q87.1 |
| Holt-Oram Syndrome | Q87.2 |
| Marfan Syndrome | Q87.4 |
| CHARGE Syndrome | Q87.8 |
| Trisomy 13 | Q91.* |
| Trisomy 18 | Q91.* |
| Wolf Hirschhorn Syndrom | Q93.3 |
| Deletion of short arm of chromosome 5 (Cri-du-chat syndrome) | Q93.4 |
| Jacobsen Syndrom 11q Syndrom | Q93.5 |
| Williams Beuren Syndrom | Q93.8 |
| Malignant neoplasm | C.* |
| Congenital adrenogenital disorders associated with enzyme deficiency | E25.* |
| Paresis und plegia | G8.* |
| Hypopituitarism | E23.0 |
| Other chromosome abnormalities, not elsewhere classified | Q99.* |

Suppl. Table 3: CHD (identified by their ICD-10-GM codes) were grouped into severity groups for analysis

| CHD severity group | ICD-10-GM codes |
| --- | --- |
| mild | Q21.0, Q21.1, Q21.88, Q21.9, Q22.1, Q22.2, Q22.8, Q23.3, Q24.0, Q24.3, Q24.8, Q24.9, Q25.0, Q25.6, Q25.7, Q26.1, Q26.3, Q26.8 |
| moderate | Q20.5, Q20.8, Q20.9, Q21.2, Q22.4, Q23.0, Q23.1, Q23.2, Q23.9, Q24.4, Q24.6, Q25.1, Q25.3, Q25.4, Q25.8, Q25.9 |
| severe | Q20.0, Q20.1, Q20.3, Q20.4, Q21.3, Q21.4, Q21.80, Q22.0, Q22.5, Q22.6, Q23.4, Q24.5, Q25.5, Q26.2 |

Suppl. Table 4: Priorities of ICD-10-GM codes from highest to lowest.

**ICD-10 diagnosis**

Q23.4 Hypoplastic left heart syndrome

Q22.6 Hypoplastic right heart syndrome

Q20.4 Double inlet left ventricle

Q20.1 Double outlet right ventricle

Q26.2 Total anomalous pulmonary venous drainage

Q21.80 Pentalogy of Fallot

Q21.3 Tetralogy of Fallot

Q22.5 Ebstein´s anomaly

Q20.0 Common arterial truncus

Q22.0 Pulmonal valve atresia

Q25.5 Pulmonal atresia

Q20.3 Transposition of the great arteries

Q25.8 Other congenital malformations of other great arteries

Q25.9 Congenital malformation of great arteries, unspecified

Q24.5 Malformation of coronary vessels

Q21.2 Atrioventricular septal defect

Q23.0 Congenital stenosis of aortic valve

Q25.3 Congenital aortic stenosis

Q25.1 Coarctation of the aorta

Q25.4 Other congenital malformations of aorta

Q24.4 Congenital subaortic stenosis

Q23.2 Congenital mitral stenosis

Q23.9 Congenital malformation of aortic and mitral valves, unspecified

Q20.5 Discordant atrioventricular connection

Q20.8 Other congenital malformations of cardiac chambers and connections

Q20.9 Congenital malformation of cardiac chambers and connections, unspecified

Q22.4 Congenital tricuspid stenosis

Q24.3 Pulmonary infundibular stenosis

Q22.1 Congenital pulmonary valve stenosis

Q25.6 Congenital pulmonary stenosis

Q25.7 Other congenital malformations of pulmonary artery

Q25.0 Patent ductus arteriosus

Q21.0 Ventricular septal defect

Q24.0 Dextrocardia

Q21.1 Atrial septal defect

Q23.1 Congenital insufficiency of aortic valve

Q21.9 Congenital malformation of cardiac septum, unspecified

Q21.88 Other congenital malformations of cardiac septa

Q23.3 Congenital mitral insufficiency

Q22.2 Congenital pulmonary valve insufficiency

Q22.8 Other congenital malformations of tricuspid valve

Q24.8 Other specified congenital malformations of heart

Q24.9 Congenital malformation of heart, unspecified

Suppl. Table 5: Description of our cohort subdivided into congenital heart defect (CHD) severity groups (mild, moderate, severe) with respective measurements of body height, weight, BMI, and head circumference of each subject, as well as gestational age and birth measurements including indication of appropriateness of weight and height for gestational age (SGA= small, AGA= appropriate, LGA= large for gestational age) and neonates with low birth weight (LBW).

|  | [ALL] N=17834 | mild N=15904 | moderate N=1328 | severe N=602 | p.overall | N |
| --- | --- | --- | --- | --- | --- | --- |
| sex: |  |  |  |  | <0.001 | 17834 |
| female | 8531 (47.8%) | 7788 (49.0%) | 503 (37.9%) | 240 (39.9%) |  |  |
| male | 9303 (52.2%) | 8116 (51.0%) | 825 (62.1%) | 362 (60.1%) |  |  |
| birth date | 2010 (6.63) | 2010 (6.58) | 2009 (7.17) | 2011 (6.53) | <0.001 | 17834 |
| gestational age | 36.7 (4.29) | 36.2 (4.60) | 37.7 (3.16) | 38.3 (2.51) | <0.001 | 2206 |
| maturity: |  |  |  |  | <0.001 | 2206 |
| fullterm (>=37) | 1474 (66.8%) | 1020 (62.3%) | 220 (78.6%) | 234 (81.0%) |  |  |
| preterm (<37) | 732 (33.2%) | 617 (37.7%) | 60 (21.4%) | 55 (19.0%) |  |  |
| sga or lbw: |  |  |  |  | 0.039 | 1225 |
| no | 1063 (86.8%) | 754 (88.4%) | 146 (82.5%) | 163 (83.6%) |  |  |
| yes | 162 (13.2%) | 99 (11.6%) | 31 (17.5%) | 32 (16.4%) |  |  |
| birth height | 47.5 (5.59) | 47.0 (6.01) | 48.5 (4.15) | 48.8 (3.82) | <0.001 | 1808 |
| birth height sds | -0.64 (1.13) | -0.53 (1.09) | -0.97 (1.18) | -0.84 (1.16) | <0.001 | 1226 |
| birth height eval: |  |  |  |  | 0.062 | 1226 |
| SGA | 138 (11.3%) | 82 (9.61%) | 27 (15.3%) | 29 (14.8%) |  |  |
| AGA | 1081 (88.2%) | 765 (89.7%) | 150 (84.7%) | 166 (84.7%) |  |  |
| LGA | 7 (0.57%) | 6 (0.70%) | 0 (0.00%) | 1 (0.51%) |  |  |
| birth weight | 2.84 (0.95) | 2.76 (1.01) | 3.04 (0.75) | 3.08 (0.68) | <0.001 | 1933 |
| birth weight sds | -0.35 (1.10) | -0.30 (1.08) | -0.43 (1.14) | -0.47 (1.13) | 0.065 | 1300 |
| birth weight eval: |  |  |  |  | . | 1300 |
| LBW | 80 (6.15%) | 51 (5.70%) | 14 (7.49%) | 15 (6.88%) |  |  |
| NBW | 1197 (92.1%) | 826 (92.3%) | 171 (91.4%) | 200 (91.7%) |  |  |
| HBW | 23 (1.77%) | 18 (2.01%) | 2 (1.07%) | 3 (1.38%) |  |  |
| birth head girth | 32.7 (3.71) | 32.3 (4.11) | 33.7 (2.36) | 33.3 (2.05) | <0.001 | 1359 |
| birth head girth sds | -0.53 (1.15) | -0.35 (1.06) | -0.75 (1.29) | -1.02 (1.19) | <0.001 | 936 |
| mother height | 167 (7.22) | 167 (7.22) | 166 (7.27) | 166 (6.95) | 0.020 | 3525 |
| father height | 179 (8.00) | 179 (8.05) | 179 (7.39) | 179 (8.20) | 0.250 | 3462 |
| target height | 173 (8.87) | 173 (8.87) | 173 (8.90) | 174 (8.85) | 0.368 | 3451 |
| target height sds | -0.16 (0.93) | -0.14 (0.93) | -0.28 (0.91) | -0.27 (0.91) | 0.014 | 3451 |
| final age | 17.8 (2.06) | 17.5 (1.33) | 19.3 (3.77) | 19.4 (4.14) | <0.001 | 1165 |
| final height | 168 (8.48) | 167 (8.07) | 170 (10.2) | 167 (10.6) | 0.014 | 1150 |
| final height sds | -0.01 (1.12) | 0.05 (1.11) | -0.22 (1.10) | -0.62 (1.19) | <0.001 | 1150 |
| final weight | 63.3 (13.6) | 63.1 (13.4) | 66.1 (15.4) | 59.7 (11.6) | 0.010 | 1162 |
| final weight sds | -0.10 (1.27) | -0.05 (1.21) | -0.24 (1.47) | -0.86 (1.52) | <0.001 | 1162 |
| visit count | 4.85 (8.19) | 4.54 (7.94) | 6.34 (7.43) | 9.95 (12.9) | <0.001 | 17834 |
| min age | 5.41 (5.51) | 5.45 (5.43) | 5.89 (6.31) | 3.12 (5.37) | <0.001 | 17819 |
| max age | 7.61 (5.77) | 7.46 (5.65) | 9.56 (6.31) | 7.25 (6.71) | <0.001 | 17819 |
| duration | 2.20 (3.34) | 2.01 (3.18) | 3.67 (3.82) | 4.13 (4.63) | <0.001 | 17819 |

Suppl. Table 6:

Description of children in our cohort with univentricular hearts (UVH), transposition of the great arteries (TGA), tetralogy of Fallot (TOF), coarctation of the aorta (COA) and atrioventricular septal defect (AVSD) with respective measurements of body height, weight, BMI, and head circumference of each subject, as well as gestational age and birth measurements including indication of appropriateness of weight and height for gestational age (SGA= small, AGA= appropriate, LGA= large for gestational age) and neonates with low birth weight (LBW).

|  | [ALL] N=870 | AVSD N=253 | COA N=171 | TGA N=178 | TOF N=144 | UVH N=124 | p.overall | N |
| --- | --- | --- | --- | --- | --- | --- | --- | --- |
| sex: |  |  |  |  |  |  | <0.001 | 870 |
| female | 357 (41.0%) | 139 (54.9%) | 49 (28.7%) | 49 (27.5%) | 78 (54.2%) | 42 (33.9%) |  |  |
| male | 513 (59.0%) | 114 (45.1%) | 122 (71.3%) | 129 (72.5%) | 66 (45.8%) | 82 (66.1%) |  |  |
| birth date | 2011 (6.48) | 2012 (5.96) | 2010 (6.67) | 2010 (6.12) | 2010 (7.91) | 2011 (5.53) | 0.004 | 870 |
| gestational age | 37.9 (2.97) | 37.1 (3.52) | 37.6 (3.19) | 38.8 (2.16) | 37.9 (2.95) | 38.2 (2.39) | 0.005 | 367 |
| maturity: |  |  |  |  |  |  | 0.282 | 367 |
| fullterm (>=37) | 288 (78.5%) | 70 (72.9%) | 49 (76.6%) | 66 (86.8%) | 52 (78.8%) | 51 (78.5%) |  |  |
| preterm (<37) | 79 (21.5%) | 26 (27.1%) | 15 (23.4%) | 10 (13.2%) | 14 (21.2%) | 14 (21.5%) |  |  |
| sga or lbw: |  |  |  |  |  |  | 0.414 | 234 |
| no | 196 (83.8%) | 45 (83.3%) | 38 (84.4%) | 40 (90.9%) | 34 (75.6%) | 39 (84.8%) |  |  |
| yes | 38 (16.2%) | 9 (16.7%) | 7 (15.6%) | 4 (9.09%) | 11 (24.4%) | 7 (15.2%) |  |  |
| birth height | 48.6 (4.27) | 48.7 (4.59) | 47.3 (4.16) | 50.1 (3.00) | 47.5 (5.13) | 49.2 (3.12) | 0.003 | 304 |
| birth height sds | -0.88 (1.18) | -0.88 (1.29) | -1.13 (1.10) | -0.46 (1.04) | -1.18 (1.26) | -0.76 (1.06) | 0.024 | 234 |
| birth height eval: |  |  |  |  |  |  | 0.370 | 234 |
| SGA | 36 (15.4%) | 9 (16.7%) | 7 (15.6%) | 3 (6.82%) | 10 (22.2%) | 7 (15.2%) |  |  |
| AGA | 197 (84.2%) | 45 (83.3%) | 38 (84.4%) | 40 (90.9%) | 35 (77.8%) | 39 (84.8%) |  |  |
| LGA | 1 (0.43%) | 0 (0.00%) | 0 (0.00%) | 1 (2.27%) | 0 (0.00%) | 0 (0.00%) |  |  |
| birth weight | 3.01 (0.73) | 2.98 (0.78) | 2.90 (0.70) | 3.33 (0.59) | 2.82 (0.81) | 3.02 (0.60) | 0.001 | 341 |
| birth weight sds | -0.54 (1.11) | -0.48 (1.32) | -0.54 (0.88) | -0.18 (1.20) | -0.92 (1.09) | -0.59 (0.88) | 0.021 | 261 |
| birth weight eval: |  |  |  |  |  |  | 0.193 | 261 |
| LBW | 20 (7.66%) | 6 (10.3%) | 1 (2.13%) | 4 (7.41%) | 7 (14.3%) | 2 (3.77%) |  |  |
| NBW | 239 (91.6%) | 51 (87.9%) | 46 (97.9%) | 49 (90.7%) | 42 (85.7%) | 51 (96.2%) |  |  |
| HBW | 2 (0.77%) | 1 (1.72%) | 0 (0.00%) | 1 (1.85%) | 0 (0.00%) | 0 (0.00%) |  |  |
| birth head girth | 33.4 (2.34) | 33.7 (2.49) | 33.4 (2.65) | 33.5 (1.86) | 32.8 (2.39) | 33.3 (2.10) | 0.494 | 245 |
| birth head girth sds | -0.95 (1.27) | -0.75 (1.31) | -0.80 (1.53) | -1.14 (1.26) | -1.15 (1.09) | -0.94 (1.15) | 0.526 | 189 |
| mother height | 166 (7.17) | 166 (7.30) | 166 (7.08) | 167 (6.08) | 165 (8.13) | 165 (7.16) | 0.907 | 273 |
| father height | 179 (7.87) | 178 (7.11) | 179 (8.36) | 179 (7.46) | 178 (10.6) | 180 (6.95) | 0.812 | 264 |
| target height | 173 (8.84) | 172 (8.93) | 175 (8.07) | 177 (8.15) | 172 (9.54) | 174 (8.66) | 0.029 | 263 |
| target height sds | -0.26 (0.91) | -0.26 (0.87) | -0.21 (0.96) | -0.24 (0.85) | -0.39 (1.17) | -0.25 (0.76) | 0.929 | 263 |
| final age | 19.9 (4.59) | 19.2 (5.04) | 20.2 (4.23) | 18.3 (1.37) | 21.3 (6.16) | 19.0 (1.87) | 0.436 | 59 |
| final height | 168 (11.0) | 166 (10.7) | 174 (11.9) | 168 (12.5) | 164 (6.75) | 175 (12.7) | 0.037 | 59 |
| final height sds | -0.40 (1.26) | -0.21 (1.31) | 0.03 (1.08) | -0.31 (1.04) | -0.93 (1.04) | 0.10 (1.87) | 0.190 | 59 |
| final weight | 62.1 (12.3) | 61.0 (11.3) | 70.4 (15.7) | 57.1 (13.2) | 59.9 (8.77) | 67.1 (11.6) | 0.077 | 59 |
| final weight sds | -0.58 (1.36) | -0.38 (1.44) | -0.15 (1.44) | -1.08 (1.54) | -0.65 (1.02) | -0.40 (1.70) | 0.574 | 59 |
| visit count | 8.91 (10.4) | 7.94 (6.42) | 7.52 (7.86) | 8.29 (9.31) | 11.1 (15.9) | 11.1 (12.8) | 0.001 | 870 |
| min age | 3.08 (5.18) | 1.96 (3.83) | 3.85 (5.42) | 3.46 (5.06) | 4.11 (7.17) | 2.55 (4.20) | <0.001 | 865 |
| max age | 7.34 (6.34) | 6.58 (5.14) | 7.73 (6.31) | 7.53 (6.42) | 8.17 (8.03) | 7.11 (6.16) | 0.135 | 865 |
| duration | 4.26 (4.41) | 4.62 (3.92) | 3.88 (4.29) | 4.07 (4.47) | 4.07 (4.99) | 4.56 (4.69) | 0.385 | 865 |

Suppl. Table 7: Description of our cohort of children with Trisomy 21 (T21) subdivided into children with (T21/CHD) and without (T21/noCHD) congenital heart defect with respective measurements of body height, weight, BMI, and head circumference of each subject, as well as gestational age and birth measurements including indication of appropriateness of weight and height for gestational age (SGA= small, AGA= appropriate, LGA= large for gestational age) and neonates with low, normal and high birth weight (LBW, NBW, HBW).

|  | [ALL] N=757 | T21/CHD N=235 | T21/no-CHD N=522 | p.overall | N |
| --- | --- | --- | --- | --- | --- |
| sex: |  |  |  | 1.000 | 757 |
| female | 370 (48.9%) | 115 (48.9%) | 255 (48.9%) |  |  |
| male | 387 (51.1%) | 120 (51.1%) | 267 (51.1%) |  |  |
| birth date | 2009 (8.10) | 2010 (8.84) | 2009 (7.69) | 0.014 | 757 |
| gestational age | 37.8 (2.48) | 37.1 (2.78) | 38.0 (2.35) | 0.005 | 428 |
| maturity: |  |  |  | 0.265 | 428 |
| fullterm (>=37) | 332 (77.6%) | 73 (73.0%) | 259 (79.0%) |  |  |
| preterm (<37) | 96 (22.4%) | 27 (27.0%) | 69 (21.0%) |  |  |
| sga or lbw: |  |  |  | 0.234 | 271 |
| no | 235 (86.7%) | 59 (81.9%) | 176 (88.4%) |  |  |
| yes | 36 (13.3%) | 13 (18.1%) | 23 (11.6%) |  |  |
| birth height | 47.8 (3.77) | 46.9 (4.72) | 48.1 (3.28) | 0.033 | 315 |
| birth height sds | -0.95 (0.97) | -1.07 (1.08) | -0.91 (0.92) | 0.251 | 272 |
| birth height eval: |  |  |  | 0.118 | 272 |
| SGA | 30 (11.0%) | 12 (16.7%) | 18 (9.00%) |  |  |
| AGA | 242 (89.0%) | 60 (83.3%) | 182 (91.0%) |  |  |
| birth weight | 2.88 (0.69) | 2.75 (0.73) | 2.93 (0.67) | 0.036 | 323 |
| birth weight sds | -0.51 (1.02) | -0.60 (1.04) | -0.48 (1.01) | 0.402 | 279 |
| birth weight eval: |  |  |  | 0.242 | 279 |
| LBW | 19 (6.81%) | 8 (10.7%) | 11 (5.39%) |  |  |
| NBW | 259 (92.8%) | 67 (89.3%) | 192 (94.1%) |  |  |
| HBW | 1 (0.36%) | 0 (0.00%) | 1 (0.49%) |  |  |
| birth head girth | 32.5 (2.56) | 31.8 (2.73) | 32.9 (2.42) | 0.014 | 186 |
| birth head girth sds | -1.11 (1.19) | -1.47 (1.21) | -0.97 (1.16) | 0.016 | 166 |
| mother height | 166 (6.77) | 166 (7.12) | 167 (6.66) | 0.624 | 422 |
| father height | 178 (7.77) | 178 (8.03) | 178 (7.70) | 0.777 | 412 |
| target height | 173 (8.77) | 172 (8.87) | 173 (8.75) | 0.540 | 411 |
| target height sds | -0.25 (0.90) | -0.27 (0.90) | -0.24 (0.90) | 0.720 | 411 |
| final age | 19.7 (3.45) | 20.6 (4.58) | 19.1 (2.38) | 0.092 | 88 |
| final height | 151 (9.82) | 155 (11.0) | 149 (8.39) | 0.014 | 86 |
| final height sds | -2.98 (1.12) | -2.85 (1.25) | -3.06 (1.04) | 0.424 | 86 |
| final weight | 61.9 (13.6) | 63.6 (16.1) | 60.9 (11.7) | 0.399 | 88 |
| final weight sds | -0.60 (1.49) | -0.79 (1.59) | -0.48 (1.43) | 0.351 | 88 |
| visit count | 11.9 (14.1) | 12.9 (18.8) | 11.5 (11.4) | 0.319 | 757 |
| min age | 2.76 (5.10) | 3.72 (6.25) | 2.33 (4.43) | 0.002 | 754 |
| max age | 8.47 (6.56) | 9.00 (7.71) | 8.24 (5.97) | 0.177 | 754 |
| duration | 5.71 (5.04) | 5.28 (4.76) | 5.91 (5.15) | 0.104 | 754 |
